# EHMTI-0366. Efficacy of cognitive behavior therapy and gestalttherapy on poor sleep quality among college female students with headache

**DOI:** 10.1186/1129-2377-15-S1-J13

**Published:** 2014-09-18

**Authors:** S Asadnia, F Sepehrian Azar, N Torabzadeh

**Affiliations:** 1 Psychology Department, Urmia University of Medical Sciences, Urmia, Iran; 2 Psychology Department, Urmia University, Urmia, Iran

## Introduction

The concomitance of sleep and headaches has been known for centuries, but the details of that concomitance were mysterious. Headache and sleep disorders are common in the general population and often coexist in the same patient.

## Aims

The present study evaluated the effects of an 8-week cognitive behavior therapy and Gestalt therapy on poor sleep qualityamong 21 college female students with headache (TTH). They were randomly placed in either a control or experimental groups.

## Methods

Experimental groups were randomly divided in to Gestalt therapy and CBT groups. General design of this study was an experimental one with pre-test, post-test and control groups. First, The Pittsburgh Sleep Quality Index (PSQI) was administrated to 3 groups.Second, Cognitive behavior groups participated in CBT sessions and Gestalt therapy groups participated in Gestalt therapy sessions. Third, all participants (3 groups) completed the PSQI again after intervention and 4 weeks later. Analysis of the data involved both descriptive and inferential statistics including mean, standard deviation, Cronbach, t-test, ANOVA, and Analysis of co-variance.

## Results

This study observed more important differences such as phenomena like subjective sleep quality, sleeps latency, sleep duration after intervention in experimental groups. Post treatment assessment at a month later revealed significant improvement of sleep quality in 14 subjects. Furthermore, there wasn't significant difference between two experimental groups, in terms of the level of improvement of sleep quality.

## Conclusion

with respect to the results obtained from one way ANOVA and covariant analysis, it is concluded that Gestalt therapy and cognitive behavior therapy has an effect on the improvement of sleep quality of the female university students with headache.

No conflict of interest.

